# Role of IL‐17 in atopy—A systematic review

**DOI:** 10.1002/clt2.12047

**Published:** 2021-08-13

**Authors:** Maja A. Hofmann, Joachim W. Fluhr, Christoph Ruwwe‐Glösenkamp, Katarina Stevanovic, Karl‐Christian Bergmann, Torsten Zuberbier

**Affiliations:** ^1^ Department of Dermatology and Allergy Charité ‐ Universitätsmedizin Berlin Berlin Germany; ^2^ Department of Infectiology and Pulmonology Charité ‐ Universitätsmedizin Berlin Berlin Germany

**Keywords:** allergische Erkrankung, atopische Dermatitis, Atopie, Überempfindlichkeit, IL‐17

## Abstract

**Purpose of Review:**

Atopy is defined as the genetic predisposition to react with type I allergic diseases such as food‐, skin‐, and respiratory allergies. Distinct molecular mechanisms have been described, including the known Th2 driven immune response. IL‐17A (IL‐17) is mainly produced by Th17 cells and belongs to the IL‐17 family of cytokines, IL‐17A to F. While IL‐17 plays a major role in inflammatory and autoimmune disorders, more data was published in recent years elucidating the role of IL‐17 in allergic diseases. The present study aimed to elaborate specifically the role of IL‐17 in atopy.

**Methods:**

A systematic literature search was conducted in MEDLINE, Embase, and Cochrane Central Register of Controlled Trials, regarding IL‐17 and atopy/allergic diseases.

**Results:**

In total, 31 novel publications could be identified (food allergy *n* = 3, allergic asthma *n* = 7, allergic rhinitis [AR] *n* = 10, atopic dermatitis [AD] *n* = 11). In all allergic diseases, the IL‐17 pathway has been investigated. Serum IL‐17 was elevated in all allergic diseases. In AR, serum and nasal IL‐17 levels correlated with the severity of the disease. In food allergies, serum IL‐17E was also elevated in children. In AD, there is a trend for higher IL‐17 values in the serum and skin specimen, while it is more expressed in acute lesions. In allergic asthma, serum IL‐17 levels were increased. In two studies, higher serum IL‐17 levels were found in severe persistent asthmatic patients than in intermittent asthmatics or healthy controls. Only one therapeutic clinical study exists on allergic diseases (asthma patients) using a monoclonal antibody against the IL‐17 receptor A. No clinical efficacy was found in the total study population, except for a subgroup of patients with (post‐bronchodilator) high reversibility.

**Summary:**

The role of IL 17 in the pathogenesis of allergic diseases is evident, but the involvement of the Th17 cytokine in the pathophysiological pathway is not conclusively defined. IL‐17 is most likely relevant and will be a clinical target in subgroups of patients. The current data indicates that IL‐17 is elevated more often in acute and severe forms of allergic diseases.

## 1 INTRODUCTION

**FIGURE 1:**
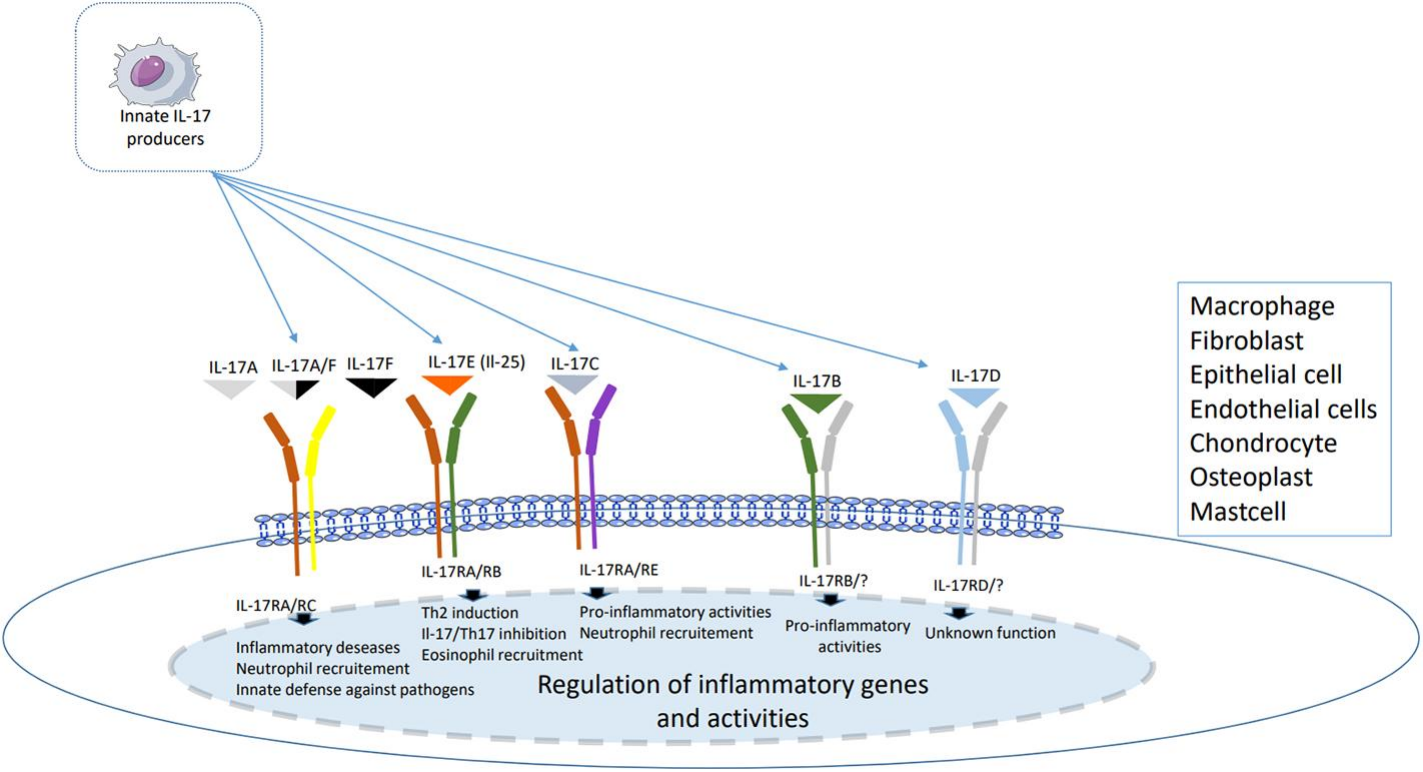
PRISMA diagram showing the process of study selection

Interleukin‐17 is a pleiotropic cytokine, which belongs to the IL‐17 family. So far, six members have been described in the interleukin 17 (IL‐17) family (Figure 1): IL‐17A (commonly known as IL‐17), IL‐17B, IL‐17C, IL‐17D, IL‐17E (also known as IL‐25), and IL‐17F.^1^ IL‐17A and F are the closest members, with 50% homology.^2^

T‐helper (Th)‐17 cells, and their major cytokine IL‐17, have been attributed to several inflammatory disorders, namely psoriasis, arthritis, and inflammatory bowel disease.^3^ Th1‐, Th2‐, and Th17‐cells are important for eradicating intracellular pathogens, helminths, as well as extracellular bacteria and fungi, respectively.^4^

IL‐17 can act on multiple cell types to induce the production of pro‐inflammatory cytokines, chemokines, and antimicrobial peptides (AMPs).^5^ Pro‐inflammatory cytokine production, such as IL‐1, IL‐6, IL‐8, matrix metalloproteinases, and TNF‐alpha, can be induced via IL‐17 receptors. New drugs such as secukinumab, brodalumab, or ixekizumab, targeting IL‐17 and IL‐17 receptors have been licensed as systemic drugs for psoriasis and other autoinflammatory diseases.

Atopic allergic inflammation includes asthma, allergic rhinitis (AR) and conjunctivitis, atopic eczema, and food allergies. Th2‐mediated immune pathway plays a major role in allergic immune responses. The permanent exposure of allergens induces the recruitment of different cell types, for example, mast cells, dendritic cells, and eosinophils, initiating cytokine production and resulting in the onset of clinical allergic symptoms. Both Th1‐ and Th2‐cells are involved in the onset and progression of allergic inflammation.^6^

Besides the classical Th1/Th2‐concept, other pathways and cytokines have been characterized, which may play a crucial role in hypersensitivity diseases.

As a pro‐inflammatory cytokine, it is of interest to identify the role of IL‐17 in the pathogenesis of different allergic diseases. In recent years, several new studies have investigated the involvement of Th17 cells and IL‐17 in allergic diseases.

The present literature review aimed to evaluate the role of the IL‐17 family in atopic allergic inflammation.

## 2 METHODS

**FIGURE 2:**
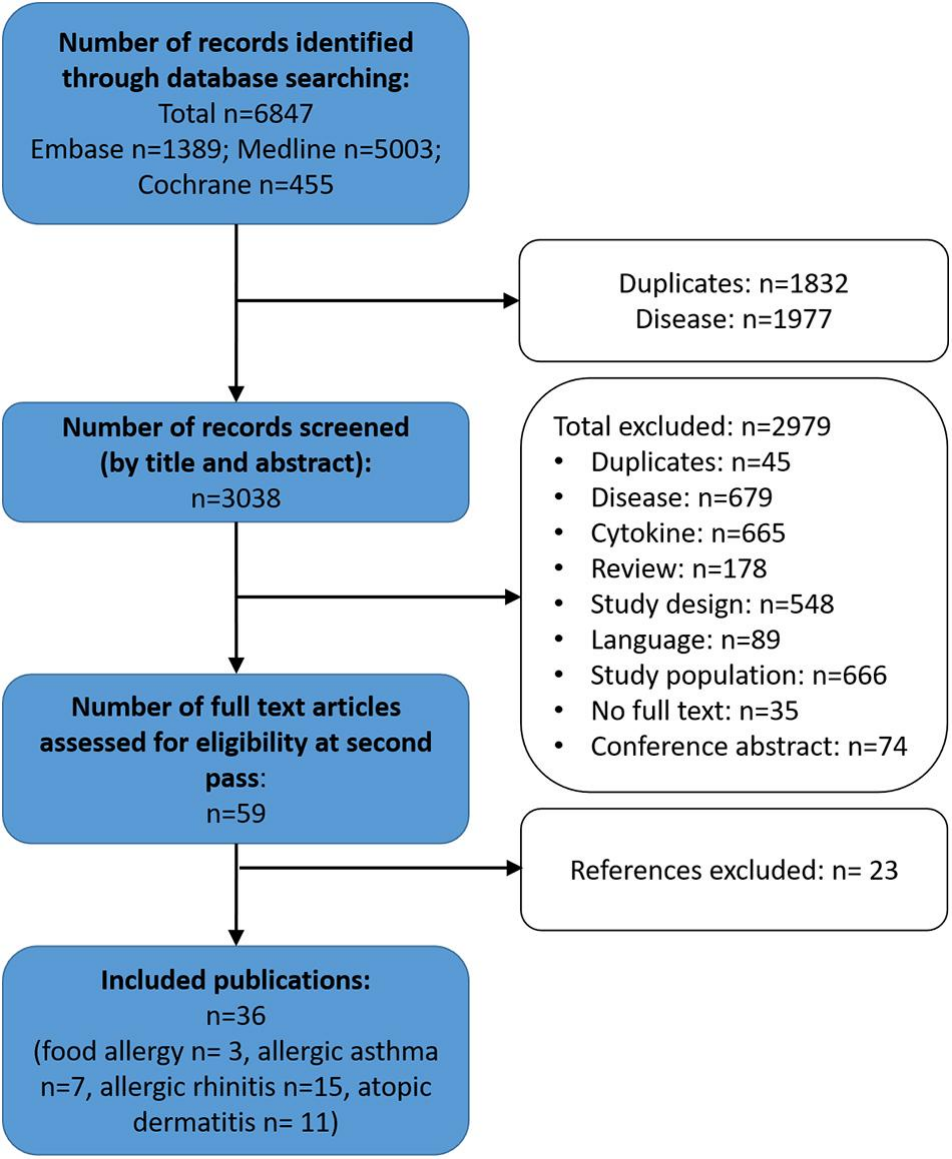
Regulation of inflammatory genes and activities

A systematic literature search was performed in Medline, Embase, and Cochrane Central Register of Controlled Trials (Figure 2) until January 31st, 2021, following the PRISMA guidelines. The main search terms were IL‐17, IL17, or interleukin 17, and atopy, allergic disease, allergic skin disease, allergic dermatitis, atopic eczema, atopic dermatitis (AD), allergic asthma, atopic asthma, AR, atopic rhinitis, and food allergies. A total of 6600 titles and abstracts were retrieved. After removing duplicates, 2994 titles and abstracts were screened. A manual search was also performed on the reference section of the included studies to identify additional relevant studies. All case‐control studies that compared IL‐17 serum levels of food allergic patients, patients with AR, allergic asthma, and atopic eczema with healthy controls were included. Additionally, case‐controlled studies involving AR measuring IL‐17 levels in the nasal fluid, as well as atopic eczema studies examining skin specimen were included. Studies were excluded from analysis due to multiple reasons, such as diseases (e.g., non atopic allergies), other cytokines than IL17, reviews, unclear study design, unclear study population, language other than English, conference abstract only and no full‐text availability. A standardized data‐collection protocol was followed to gather the data regarding publication year, number of patients, number of controls, and method of IL‐17 measurement.

In total, 31 publications were selected, a summary of the main findings is shown in Table 1.

**TABLE 1:** Main findings

Main findings
Elevated IL‐17 serum values in adults and children with food allergies, but less studies
Severe asthma associated with higher IL‐17 levels, but IL‐17 antibody showed no efficacy in phase III study
Correlation of IL‐17 values with severity of disease in allergic rhinitis
In AD, IL‐17 increase in acute disease

## 3 RESULTS

### 3.1 Food allergy

In total, 103 citations were found for food allergy and IL‐17. Three studies with a total of 348 patients (300 children) and 32 controls (seven children) were selected.

One study with 30 adult patients with an elevated concentration of the allergen‐specific IgE showed that serum IL‐17A concentrations were higher in patients with food allergic hypersensitivity (1.24 pg/ml ± 0.63) compared to healthy volunteers (0.87 pg/ml ± 0.41).^7^

Another study investigated the role of IL‐17A and IL‐17E also measured by ELISA in children with allergic diseases (AD, hay fever, and asthma) in patients with food allergies. They detected that increased IgE levels specific to food allergens were positively related to IL‐17E serum concentrations. Median and interquartile ranges for IL‐17E were 80 pg/ml (31.6–208.8) and for IL‐17A 267.8 pg/ml (164.1–399.4). IL‐17A showed the tendency to be negatively associated with allergic sensitization, especially in some food allergens, but specific Th17 responses were not assessed. It was stated that a dysregulation of the IL‐17E/IL‐17A axis might have the same impact as the dysbalance in the Th1/Th2 axis regarding the allergic outcome.^8^

These findings were verified in a small study investigating the role of the Th17 response, which included 18 children with a peanut allergy and to at least one additional food allergen, and eight atopic adults without food allergies. They found an impaired Th17 response in the children but not in the atopic patients without food allergy. They stated that the low IL‐17 production could allow for an aberrant Th2 response, resulting in progression of the allergies.^9^

### 3.2 Allergic asthma

In total, 540 citations were found that explored the involvement of IL‐17 in allergic asthma. Seven studies were selected in which six studies examined the serum/plasma level of IL‐17 of asthmatic patients measured by ELISA technology, and one study analysed Th17 cells in the blood of asthmatic patients. Studies measuring IL‐17 level in bronchial biopsies were excluded.

Serum IL‐17 levels were found to be elevated in all studies involving adults and children with allergic asthma.

In one study, allergic asthmatic patients showed higher plasma IL‐17 than normal controls (22.40 vs. 11.86 pg/ml), although the differences were not statistically significant.^10^ Another study with asthmatic patients and AR showed significantly elevated serum IL‐17 levels in patients during asthma attacks and remission, compared with healthy control subjects.^11^

Chien et al.^12^ found that in asthmatic patients with different severities, serum IL‐17 levels were significantly higher in mild to severe persistent asthmatic patients than in intermittent asthmatics or healthy controls. Serum IL‐17 levels were higher in the uncontrolled (mean 52.33 pg/ml) and partly controlled groups (12.67 pg/ml) than in the controlled group (5.46 pg/ml). In accordance with FeNO levels, a significantly higher proportion of mild to severe persistent asthmatics had higher serum IL‐17 values than mild intermittent asthmatics. Also, in asthmatic children, elevated serum IL‐17 could be detected (79.5 pg/ml), while for children with asthma and AR, higher serum values were measured (107.3 pg/ml).^13^

In a study analyzing serum IL‐17F levels, the results showed that IL‐17F was significantly up‐regulated in asthmatic patients. All patients had a wide IL‐17F range level of 71.40–1904 pg/ml (916.94 ± 520).^14^

An investigation of the role of plasma IL‐17 in obese asthma patients in both acute and stable settings divided stable asthma subjects into well‐controlled, partly controlled and poorly controlled asthma, and compared them with individuals with acute asthma, for plasma IL‐17 levels. The results of this study indicated that plasma IL‐17 concentrations steadily increased as the asthma control level decreased, with the highest levels of IL‐17 values observed in acute asthma 21.8 pg/ml (9.7, 50.5), respectively. Furthermore they investigated the IL‐17 levels depending on the BMI index, lean (18.5 kg/m^2^ ≥ BMI < 25 kg/m^2^), overweight (25 kg/m^2^ ≥ BMI < 30 kg/m^2^) and obese (BMI ≥ 30 kg/m^2^). Plasma IL‐17 levels were the highest in the obese asthmatic group (13.1 pg/ml) compared with the lean asthmatic group (8.1 pg/ml).^15^

The percentage of Th17 cells in the blood was significantly increased in patients with allergic asthma (1.53 ± 0.30%) than that of the control group (0.97 ± 0.23%).^16^

Collectively, existing clinical data shows increased levels of IL‐17 in severe asthma. However, attempts to block IL‐17 in asthmatics as a therapeutic target have been disappointing so far. Currently, there is only one randomized, placebo‐controlled study using brodalumab, a human anti‐IL‐17 receptor monoclonal antibody. In patients with moderate to severe asthma, there was no effect over 12 weeks on clinical symptoms, and FEV1 in the asthmatic patients was observed.^17^

### 3.3 Allergic rhinitis

In total, 210 articles were reviewed and were screened by title and abstract, and 15 publications were assessed for eligibility in full text, while five records were excluded (two of which were in the Chinese language, two were without a report on relevant outcomes, and one was using mice models). Studies that included serum and nasal fluid were included in the review. In 10 studies, serum IL‐17 levels were examined by ELISA, and in two studies, nasal fluid IL‐17 levels were examined.

In all studies, elevated IL‐17 in serum or nasal fluid could be shown. The mean serum and nasal IL‐17 levels were higher in AR (107.7 ± 79.61 and 527.36 ± 738.7 pg/ml) than in the control group (76.29 ± 28.94 and 328.9 ± 430.8 pg/ml).^18^ In patients with AR, elevated IL‐17, IL‐22, and TGF‐*β* serum levels were detected compared with a control group.^19^ Serum IL‐17 levels and Th17‐cells were increased in AR patients and showed an upregulation with the severity of total nasal symptoms.^20^ In birch‐monosensitized patients, IL‐17 levels also correlated with clinical symptom severity.^21^

In another study, serum IL‐17 levels in AR and control groups were 668.55 ± 45.15 and 573.53 ± 17.42 pg/ml, respectively. Furthermore, they showed that a specific subgroup of T‐cells, ɤδ T‐cells, are associated with the secretion of IL‐17.^22^

In children with AR, asthma, or both, the highest IL‐17 levels were found in children with AR and asthma. IL‐17 was significantly higher in AR, Asthma, and both, compared to a control group (79.5 ± 17.7 g/ml Asthma, 71.3 ± 24.6 pg/ml AR, 107.3 ± 22.4 pg/ml Asthma and AR, 21.8 ± 6.5 pg/ml control).

Another study investigated serum IL‐17 levels in patients with mild‐moderate and severe AR. They found significantly elevated cytokine levels in both subgroups compared to healthy volunteers. The highest levels of IL‐17 were detected in the severe group of AR patients. Furthermore, all AR patients showed decreased serum IL‐17 and total IgE levels after 6 months of cluster immunotherapy.^23^

A recent paper showed elevated IL‐17 cytokine serum levels in 88 patients with AR compared to 88 healthy controls. The elevated Il‐17 serum level was associated with elevated ECP‐ and IL‐33 levels.^24^ These markers might be useful to monitor disease activity.

### 3.4 Atopic dermatitis

In total, 4133 articles were reviewed and were screened by title and abstract. We assessed 22 publications for eligibility in full text, while 11 records were excluded (four were in the Chinese language, three had no relevant outcomes reported, four were mice models). For AD, studies examining skin biopsies were also included. From the 22 publications, 11 studies could be selected. In eight studies, patients' skin specimen were analysed, and in six studies, TH17 cells in the blood or IL‐17 serum levels were evaluated.

In AD, few studies dealt with the serum IL‐17 level in patients.

One study measured serum IL‐17 levels by ELISA and found higher levels in the AD group (10.47 ± 3.39 pg/ml) compared with the control group (9.63 ± 3.36 pg/ml), but this difference was not statistically significant.^25^ Another study on 49 children with AD versus control, measured IL‐17 mRNA levels by real‐time quantitative reverse transcriptasepolymerase chain reaction in PBMCs (6.87 pg/ml [5.62–7.95] vs. 1.68 [1.40–2.07]), and in the serum by ELISA (32.60 [28.55–37.85] pg/ml vs. 11.45 [9.78–12.55] pg/ml). In both sample types, IL‐17 levels were significantly higher in AD patients than healthy controls.^26^ In 181 children with AD, serum IL‐17 levels were significantly higher compared to healthy children. Furthermore, SCORAD‐levels and IL‐17 showed a positive correlation.^27^ In the peripheral blood of AD patients, Th17 cells, as well as IL‐17 and IL‐23 levels were found to be significantly higher compared to patients with allergic contact dermatitis versus healthy patients, but lower than those of psoriasis patients.^28^

Regarding different stages of AD, it was reported that the percentage of IL‐17 positive lymphocytes in the blood of AD patients was significantly higher in acute versus chronic atopic eczema. Furthermore, the number of circulating Th17‐cells correlated with the clinical severity of atopic eczema.^29^

In a small study with AD and psoriasis patients, Nograles et al.^30^ showed that Th17 cells were significantly decreased in AD skin specimens compared to psoriatic skin, but in peripheral blood there was no difference. IL‐17 mRNA was reported to be 20‐fold decreased in AD skin, contrasted by only a 2‐fold decrease in Th17 cell levels.

A comparison of AD and psoriasis in erythrodermic patient revealed no significant difference for Th17 cells between these two diseases in histological skin specimens.^31^ Another immunohistological study in patients with atopic eczema revealed that IL‐17 can be expressed as a function of different disease stages. IL‐17 was significantly increased in acute lesions compared to chronic lesions and healthy skin. Furthermore, they showed that IL‐17—in combination with TGF‐beta—could be responsible for the development of tissue fibrosis in skin lesions of atopic eczema.^32^

Recently, Gamez et al.^33^ reported reduced IL‐17 and IL‐25 in cord blood in seven infants who developed AD during the first 12 months after birth compared to 24 infants who did not develop AD. This data highlights the in‐utero period as being critical for potential maternal influence for the development of AD in infants.

## 4 DISCUSSION

IL‐17 seems to play a role in all atopic diseases and serum values have been reported to be higher in severe stages of atopic diseases.

Regarding food allergies, the pathogenesis of food allergy is not fully understood. The main pathogenic immunological mechanism in food allergies is regarded as Th2 mediated, while the role of Th17 cells in food allergies is still being investigated. For IL‐17A and IL‐17E serum data are available. As there is only one *in vivo* human study investigating IL‐17E, it is difficult to state the impact of IL‐17E in allergic diseases. To which extent the Th17 impairment plays a role in primary food allergies has to be investigated further, and ideally not only focusing on IL‐17A. To gain a specific understanding on the different IL‐17 subsets and IL‐17R in food allergies, the regulation of these cytokines should be further elucidated.

AR is a very common allergic inflammation and probably the most prevalent form of rhinitis. About 10%–40% of AR patients have asthma comorbidity, whereas nearly all patients with asthma have moderate to severe rhinitis.^34^ After allergens are taken up and presented to T‐lymphocytes, different cytokines are released. IL‐4 and IL‐13 are mainly involved in AR. They interact with B‐lymphocytes, resulting in the production of allergen‐specific IgE with mast cell activation. As is the case with most allergic diseases, AR is known to be Th2 driven. As IL‐17 can induce IL‐8 production, this suggests an indirect effect of the Th17 pathway with secretion of IL‐17,^35^ which is also in accordance with the findings of elevated serum and nasal IL‐17 levels.

Besides the reported *in vivo* studies, mouse‐models were used to investigate the possible involvement of IL‐17 in AR. IL‐17A deficient mice showed a significant decrease in allergic symptoms.^36^ To elucidate the underlying mechanism of IL‐17 in the pathogenesis of neutrophilic AR, an LPS‐induced neutrophil‐dominant AR model was established. Neutrophil infiltration was dependent on IL‐17 and VEGF interaction. Inhibition of VEGF signaling also reduced neutrophil infiltration and IL‐17 production.^37^

In summary, the involvement of IL‐17 in the pathogenesis of AR is reliable. Increased IL‐17 serum levels might be considered as a marker of disease severity in AR patients. As one study showed a decrease of IL‐17 in patients with immunotherapy, IL‐17 could perhaps act as a serum marker under immunotherapy.

In asthmatic patients, there seems to be an impact on IL‐17. When considering approaches to pathophysiology, it should be emphasized that the term “asthma” is a clinical diagnosis encompassing a spectrum of airway obstructive inflammatory diseases. The selected studies showed that serum IL‐17 is elevated in asthma, and especially increased in severe asthma. Indeed, there are more factors that have an impact on the IL‐17 level. Increasing IL‐17 levels were seen in patients with asthma and a high BMI. In the same study, IL‐17 levels were found to be elevated in asthmatic patients with elevated depressive symptoms in obesity.

In a double IL‐17A/F knock‐out mouse model, Jirmo et al. showed a reduced influx of dendritic cells into lungs and lung‐draining lymph nodes.^38^ The authors concluded that IL‐17 enhances airway dendritic cell activation, migration, and function.

Besides the reported studies dealing with the serum and plasma levels of IL‐17, in AR there are some preclinical models showing the impact of IL‐17 in asthma. In a mouse‐model, Choy et al.^39^ showed that the simultaneous neutralization of IL‐13 and IL‐17 protected mice from eosinophilia, mucus hyperplasia and airway hyperreactivity, and subsequently abolished the neutrophilic inflammation in asthma. In a subset of patients with severe asthma, chronic airway inflammation is associated with infiltration of neutrophils, Th17 cells, and elevated expression of Th17‐derived cytokines (including IL‐17 and also IL‐21).^40^ IL‐17 is involved in neutrophil‐recruitment into the airways. Thus, some patients with severe asthma exhibit airway neutrophilic inflammation, which is induced by Th17 cells and linked to elevated IL‐17. These patients often appear to respond poorly to corticosteroid treatment and don't show an improvement of their clinical symptoms. This poor response may partially be due to the resistance of Th17 cells to corticosteroid treatments. Peripheral neutrophils from allergic asthma patients express higher IL‐17 cytokine levels than those from healthy control subjects.^41^

Regarding the different subtypes of IL‐17A, there was an experimental study revealing that stimulating asthmatic neutrophils with IL‐21, 23, and 6, enhanced the production of IL‐17A and IL‐17F at significantly higher levels compared to healthy controls.^42^ It has recently been shown that *Candida albicans* induces anti‐fungal modulations of Th17 cells, promoting pathogenic airway inflammation triggered by airborne fungi like *Aspergillus fumigatus*. This finding has important implications for the postulated gut‐lung axis.^43^

In AD, only four studies dealt with serum IL‐17 values. There seems to be an increasing value of Th17 cells and IL‐17 in skin specimen in dependency of the lesions (chronic or acute ones). AD is a disease with a wide variety of clinical courses depending on the age group. Also, different clinical courses from chronic to more acute development can be observed. The pathogenesis of AD has to be seen as a multifactorial disease with the involvement of genetics, environmental factors, impaired epidermal barrier, and a dysregulated immune system. Classically AD is Th2 driven. But AD inflammation shows a biphasic pattern, with a predominant Th2 response in the acute stage and then switching to a more Th1 driven immune response in the chronic stage. AD is usually colonized by *Staphylococcus aureus*, which is positively correlated with the disease severity. Bacteria‐derived superantigen staphylococcal enterotoxin B can trigger IL‐17 secretion; thus, microbial stimuli might activate the IL‐17 secretion in AD patients.

In addition to the *in vivo* studies, murine models have been established to elucidate the role of IL‐17 in AD. In different murine models, IL‐17 was highly expressed in the acute AD‐like skin lesions.^44^, ^45^, ^46^

These studies revealed a difference in chronic and acute AD, which might have an impact on future systemic and topical treatment approaches, however the main limitation of systematic reviews is the heterogeneity in the baseline characteristics of all included studies. Furthermore significant differences in IL‐17 levels were reported due to different measurement techniques and different commercially available ELISA kits that were used.

## 5 CONCLUSION

In conclusion, IL‐17 is an emerging cytokine regarding allergic inflammation. Many publications have shown that Th17 cells are directly and indirectly involved in allergic atopic diseases. Nevertheless, the role of IL‐17, Th17 cells, and the interaction with different types of IL‐17R has not been investigated systematically and has to be elucidated in detail. In the acute and severe phases of allergic disease, there seems to be a more prominent role of IL‐17 than in chronic phases. Based on the results of the first interventional trial in asthma patients, it is very likely that in future research, a more differentiated approach with a subgroup‐ analysis will be used. Eventually a better understanding of the IL‐17 pathway will lead to specific approaches and to more personalized medicine in allergy.

## CONFLICT OF INTERESTS

Maja A. Hofmann: Industry consulting, research grants and/or honoraria: Almirall, Asclepion, Bayer Health Care, Galderma, GSK, Leti, L'Oreal, Novartis, Novoxel, Pfizer, Pierre Fabre, Roche Posay, Sanofi. Torsten Zuberbier: Industry consulting, research grants and/or honoraria: AstraZeneca, AbbVie, ALK, Almirall, Astellas, Bayer Health Care, Bencard, Berlin Chemie, FAES, HAL, Henkel, Kryolan, Leti, Lofarma, L'Oreal, Meda, Menarini, Merck, MSD, Novartis, Pfizer, Sanofi, Sanoflore, Stallergenes, Takeda, Teva, UCB. Organizational Affiliations: Committee member, WHO‐Initiative “Allergic Rhinitis and its Impact on Asthma” (ARIA), Member of the Board, German Society for Allergy and Clinical Immunology (DGAKI), Head, European Centre for Allergy Research Foundation (ECARF)Secretary General, Global Allergy and Asthma European Network (GA^2^LEN), Member, Committee on Allergy Diagnosis and Molecular Allergology, World Allergy Organisation (WAO). Karl‐Christian Bergmann: Industry consulting, research grants and/or honoraria in the last three years: ALK, Allergopharma, Almirall, AstraZeneca, Bencard, Boehringer, Chiesi, GSK, HAL, LETI, Lofarma, Mundipharma, Novartis, Sanofi, Teva. Joachim W. Fluhr, Christoph Ruwwe‐Glösenkamp, Katarina Stevanovic: No conflicts of interest.

## AUTHOR CONTRIBUTIONS

Joachim Fluhr: Supervision, Equal, Writing‐review & editing, Supporting; Christoph Ruwwe‐Gloesenkamp: Supervision, Supporting, Writing‐review & editing, Supporting; Karl‐Christian Bergmann: Supervision, Equal; Torsten Zuberbier: Writing‐review & editing, Equal; Katarina Stevanovic: Visualization, Equal, Writing‐review & editing, Equal.

## Supporting information

Supplementary Material
