# Supplementary material for: Role of IL‐17 in atopy—A systematic review
Source: Clin Transl Allergy. 2021 Aug 13;11(6):e12047. doi: 10.1002/clt2.12047 (PMC8361814; doi:10.1002/clt2.12047)
Supplement: Supplementary file 1 — Supplementary Material [file CLT2-11-e12047-s001.docx]

|  | **Year of publication** | **Allergic disease/inclusion criteria** | Study design/Evidence level | **Patient number** | **Examined parameters** | **Outcomes** |
| --- | --- | --- | --- | --- | --- | --- |
| Food allergy | | | | | | |
| Herberth G et al. | 2010 | 6 year old children | Cohort study/  IIb | 293 children | Evaluation of serum IL17A, IL17E and specific IgE  enzyme-linked  immunosorbent assay (ELISA) | Sensitization:29%inhalant;13.6%food;33.1% other  IL17E correlating with specific IgE levels. High IL17E in children with food or inhalant allergy. IL17A negatively associated to sensitization to some allergens.  Median and interquartile ranges (IQR) for IL-17E were 80 pg/ml (31.6–208.8) and for IL-17A 267.8 pg/ml (164.1–399.4). |
| Zbikowska-Gotz M et al. | 2015 | food allergy | Case-control study/  IIIb | 30 patients + 10 healthy controls | IL-17A, from peripheral blood neutrophils. | IL-17A: higher in patients with food allergic hypersensitivity compared to healthy controls  IL17 A 1,24 pg/ml, versus 0,84 pg/ml (control group) |
| Dhuban KB et al. | 2013 | peanut allergy + min. 1 other food allergy | **Case-control study/**  **IIIb** | 18 patients, 15 healthy controls  7 atopic children (no food allergy) + 7 healthy controls | Ex vivo PBMC analysis or in vitro PBMC stimulation with analysis of proliferation and cytokine-production of antigen-responsive cells. | Significantly impaired Th17-response in food allergic children both, ex vivo and in vitro, atopy alone showed no impairment |
| **Asthma** | | | | | | |
| Hatta et al. | 2017 | atopic asthmatic  patients | **Case-control study/**  **IIIb** | 40 patients | Serum levels of soluble  IL-17F measured using commercially available ELISA | sIL-17F significantly up regulated in asthmatic patient (p < 0.0001)  sIL-17F range level 71.40–1904pg/ml (916.94 ± 520) |
| Chen et al. | 2016 | Asthma | **Cross-sectional study/**  **IIb** | 177 stable asthmatic patients  78 acute asthmatic patients | Clinical characteristics, sputum, plasma IL-17 in lean, overweight and obese patients | - IL-17 higher in acute versus stable asthma subjects, but similar among groups  - in stable disease: IL17 higher in obese patients  - plasma IL-17 positively connected to airway eosinophilia during exacerbation  Stable asthma subjects were divided into well-controlled,partly-controlled and poorly-controlled asthma and compared with individuals with acute asthma for sputum and plasma IL-17  levels. The medians (interquartile range) in the four groups were 4.0 (2.1, 6.3), 3.8 (2.5, 7.2), 6.2 (2.8, 9.6) and 11.0 (6.2, 17.6) pg/ml  respectively for sputum IL-17 and 7.6 (4.8, 15.5), 7.8 (4.6, 13.7), 14.0 (7.9, 22.5) and 21.8 (9.7, 50.5) pg/ml respectively for plasma IL-17. |
| Lv H et al | 2016 | Asthma | **Case-control study/ IIIb** | 20 asthmatic patients, 23 control | Serum IL-17 and eotaxin levels in asthmatic patients with allergic rhinitis during attacking and  remission | Serum IL-17 and eotaxin levels were  significantly elevated in patients during asthma  attack and remission (*p*< 0.05) |
| Tao B et al | 2015 | Allergic rhinitis (AR), Asthma and AR and Asthma | **Cohort study IIB** | 24 children with AR, 22 children with BA, 18 children with AR accompanying with BA, and 20 healthy controls | Cytokine expressions in plasma were determined by ELISA | pIL-17 significant higher in AR, asthma and both (p<0,05) 79,5 g/ml ±17.7 asthma, 71.3 ±24.6 pg/ml AR, 107.3±22.4 pg/ml Asthma and RA 21.8±6.5 pg/ml control |
| Chien et al. | 2013 | Mild intermittent, mild persistent and modereate to severe persistant asthma | **Case-control study/ IIIb** | 120 asthmatic children, mild intermittent  (n = 42), mild persistent (n = 42), and moderate to severe persistent (n = 36) groups, and  20 healthy controls | Plasma IL-17 levels were measured using a Human IL-  17 Quantikine ELISA kit | Serum IL-17 and FeNO levels significantly higher in mild to severe persistent asthmatic patients than in intermittent asthmatics or healthy controls (P < 0.05).  Serum IL-17 levels higher in  the uncontrolled (43.06 + 52.33 pg/mL) and partly controlled (12.52 + 12.67 pg/mL) than in the controlled group (4.85 + 5.46 pg/mL)  Mean serum IL-17 levels were high in moderate to severe persistent asthmatic subjects when compared to mild intermittent to persistent asthmatic subjects (P < 0.01) |
| Wong et al | 2001 | Allergic asthma | **Case-control study/ IIIb** | 41 allergic asthmatics and 30 health control  subjects | Plasma cytokines measured ELISA | Allergic asthmatic patients higher plasma IL-17 than normal controls (22.40 versus 11.86 pg/ml), not statistically significant (p= 0.077) |
| Li et al. | 2017 | allergic asthma |  | 35 children with allergic asthma and 35 healthy control children | Flow cytometry was used to detect Th17 and Treg cells. | The percentage of Th17 cells (CD4+IL-17+) significantly increased in patients with AA (1.53 ± 0.30%) than that of the control group (0.97 ± 0.23%) (*p <* 0.001) |
| **Allergic rhinitis** | | | | | | |
| Erkan K et al. | 2020 | moderate to severe rhinitis | **Non-randomized controlled study/**  **IIc** | 38 patients with moderate-severe AR, 36 controls | Serum levels of IL-10, IL-17 and neopterin in serum and nasal lavage fluid | Mean serum and nasal IL-17 were higher in AR (107.7 ± 79.61 and 527.36 ± 738.7 pg/ml) than the control group (76.29 ± 28.94 and 328.9 ± 430.8 pg/ml) (p < 0.05 and p > 0.05). |
| Tao B et al | 2015 | Allergic rhinitis (AR), Asthma and AR and Asthma | **Cohort study IIB** | 24 children with AR, 22 children with BA, 18 children with AR accompanying with BA, and 20 healthy controls | Cytokine expressions in plasma were determined by enzyme linked immunosorbent assay. | 79,5 pIL-17 significant higher in AR, Asthma and both (p<0,05) g/ml ±17.7 Asthma 71.3 ±24.6 pg/ml AR 107.3±22.4 pg/ml Asthma and AR 21.8±6.5 pg/ml control |
| König K et al. | 2015 | SAR and PAR | **Case control study IIb** | 44 participants suffering from SAR, 45 participants suffering  from PAR and 48 healthy subjects | Nasal secretions analysed by Bio-Plex Cytokine Assay | IL-17 levels significantly elevated in the SAR group (median 20 pg/ml, range 0–90 pg/ml; *p* < 0.001 vs. control/PAR) while the PAR group and the controls showed similar low levels (PAR: median 0 pg/ml, range 0–147 pg/ml; controls: median 2 pg/ml, range 0–320 pg/ml) |
| Bayrak Degirmenci P et al. | 2018 | Allergic rhinitis | **Case-control study/**  **IIIb** | 65 patients and 31 healthy controls | Serum levels of IL-10, IL-17, TGF-β, IFN-γ, IL-22, and IL-35 | IL-17, IL-22, and TGF-β higher in the patients,  IL-17 0.45 (1.75–0.001) 0.68 (7.18–0.11) 0.038 ng/ml |
| Tang J et al. | 2014 | Allergic rhinitis | **Case-control study/**  **IIIb** | 36 patients and 22 healthy controls | 1. Frequency of IL-22+, IL-17A+, and IL-9+T helper (Th) cells in peripheral blood. 2. IL-22 and IL-17A serum levels | Frequency of IL-17A+Th cells higher in AR patients and correlating with severity of symptoms  Serum levels of IL-17A higher (42pg/ml ) in AR patients compared to controls |
| Ciprandi G et al. | 2008 | pollen-induced allergic rhinitis | **Case-control study/**  **IIIb** | 56 patients | Serum IL-17 levels were evaluated by ELISA | Serum IL-17 levels significantly related to clinical symptoms, druguse and peripheral eosinophil counts (P= 0.0001 for all)  mean serum IL-17 value 3.11 pg/ml (SD4.36 pg/ml; SME 0.58 pg/ml). |
| Ciprandi G et al. | 2008 | Birch-monosensitized patients with no active AR | **Case-control study/**  **IIIb** | 19 patients and 8 healthy controls | Serum level of IL-17A, total and specific IgE levels, allergen threshold dose and eosinophil count. | Allergic patients exclusively sensitized to birch alone significantly higher median levels of IL-17 25.63 pg/ml median, control 0,28 pg/ml |
| Xuekun H et al. | 2014 | Allergic rhinitis | **Case-control study/**  **IIIb** | 32 patients and 20 healthy controls | 1. Percentages of gammadelta-T-cells and Th17 cells in peripheral blood 2. Serum IL-17 levels by ***ELISA*** | 1. Percentage of gammadeta-T-cells and Th17 cells in the AR group significantly higher 2. The levels of IL-17 in the AR group also significantly higher   The serum IL-17 levels in the AR and control groups were (668.55 ± 45.15 pg/ml) and (573.53 ± 17.42 pg/ml), respectively |
| Aly M et al. | 2017 | Allergic rhinitis | **Non-randomized controlled study**  **IIIb** | 13 patients with mild-to-moderate AR, 13 with severe AR, 13 healthy controls,  11 AR-patients did receive a 6-month immunotherapy. | Serum IL-17 and total IgE | Serum IL-17 levels and serum IgE significantly increased in patients with AR, significant decrease of both after immunotherapy.  Serum IL-17 level 3.1pg/ml + 1.3 (3–5) control 12.3 + 2.5 pg/ml (8.5–16) moderat 42.7 + 15 pg/ml(23–63.3) severe |
| Amin et al. | 2020 | Allergic rhinitis | **Case-control study/**  **IIIb** | 88 patients with AR, 88 healthy controls, | Serum 1L-17 level | Serum IL-17, ECP and IL-33 levels significantly increased in patients with AR |
| **Atopic dermatitis** | | | | | | |
| Vandeghinste N et al. | 2018 | Atopic dermatitis and psoriasis | **Case-control study/animal research study/**  **IIc** | 10 psoriatic patients,  10 AD patients  Wild-type mice and flaky-tail mice | Binding of IL-17C with a specific antibody (MOR106), immunhistology | immunohistochemistry analysis showed IL 17C expression in the lesional skin of psoriatic and AD patients ,IL-17Cwas increased in the keratinocytes and was also detected in infiltrating immune cells in the dermis |
| Tan et al. | 2017 | Atopic dermatitis |  | Blood samples were collected from 87 children with AD and 60 healthy control subjects. In addition, 10 skin biopsies from each group | Skin and serum expression levels of IL-17 were analyzed by immunohistochemistry  and enzyme-linked immunosorbent assay, respectively. | The frequency of IL-17+ cells was higher in the dermis of patients with AD compared with control subjects (*p* <.01). Serum IL-17 levels were higher in the AD group (10.47+ 3.39 pg/mL) compared with the control group (9.63+3.36 pg/mL), b not statistically significant (*p* = .1416). |
| Simon D | 2014 | Atopic dermatitis, irritant or allergic contact dermatitis |  | 27 patients with biopsies  biopsies taken from positive patch test (PT) reactions  to contact allergens (CPT), house  dust mite or pollen (APT), and 0.25% sodium  lauryl sulfate (IPT)  as model for ACD, AD, and ICD, | Skin specimens taken from positive patch test at days 2, 3, and 4. Inflammatory cells as  well as the expression of cytokines and extracellular matrix proteins evaluated  by immunofluorescence staining and confocal microscopy. | Expression of IL-17 was observed in all eczema subtypes. In APT, the expression  of IL-17 was highest at D2 and D3 (P = 0.036), whereas in IPT, a sharp increase was observed only at D4. |
| Toda M et al. | 2003 | Atopic dermatitis | **Case-control study/**  **IIIb** | 8 atopic dermatitis patients (acute lesion, chronic lesion and uninvolved skin from each), 8 healthy controls | TGF-beta1, IL-11, and IL-17 expression in skin biopsy. | - TGF-beta1 significantly increased in both acute and chronic lesions  - IL-11 significantly increased only in chronic lesions  - IL-17 significantly associated with acute lesions. |
| Koga C et al. | 2008 | Atopic dermatitis | **Case-control study/ IIIb** | 46 AD patients, 9 healthy controls | Th17 cells in the peripheral blood and skin lesions of AD, with a study of the effect of IL-17 on the production ofbcytokines/chemokines and vascular endothelial growth factor (VEGF) by keratinocytes | - Significant correlation between the percentages of IL-17+ and IFN-gamma+ cells - IL-17+ cells infiltration of the papillary dermis more markedly in the acute than chronic AD lesions.  - IL-17 stimulated keratinocytes to produce GM-CSF, TNF-alpha, IL-8, CXCL10, and VEGF.  - Synergistic effect between IL-17 and IL-22 on IL-8 production.  - Number of Th17 cells increased in the peripheral blood and acute lesional skin of AD  -IL-17-positive lymphocytes found in the papillary areas in the upper dermis, percentage of IL-17-bearing lymphocytes higher in the acute than in chronic lesions. |
| Ma L et al. | 2014 | Atopic dermatitis | **Case-control study/**  **IIIb** | 35 AD patients, 59 controls (8 allergic contact dermatitis, 20 psoriasis, 31 healthy subjects) | Th17 and Treg cells percentage, mRNA levels of RORγt and Foxp3, Th17- and Treg-related cytokines in different sample materials (PBMC, skin specimen, serum and PBMCs culture supernatant after recombinant Dermatophagoides pteronyssinus antigen stimulation) | Immune imbalance in Th17 and Treg cells in AD patients:  - significantly higher Th17 cells percentage, RORγt, IL-17 and IL-23 levels in peripheral circulation  - negative association between Th17 and Treg cells percentage in AD patients  - positive correlation of AD severity score with Th17 cells percentage and Th17/Treg ratio, while negatively correlated with Treg cells percentage |
| Ma L et al | 2014 | Atopic dermatitis |  | in 49 AD children (6–16 years old) and 30 age-matched  healthy controls.. | Th17 cells percentage, IL-17 mRNA  level, and IL-17 serum concentration were measured by flow  cytometric analysis, PCR, and ELISA | AD patients showed an obvious increase in Th17 cells percentage compared to healthy controls 1.60 (1.36–1.80)% vs.0.40 (0.30–0.50)%, P < 0.01]. The IL-17 mRNAlevel in PBMCs 6.87 (5.62–7.95) vs. 1.68 (1.40–2.07),P < 0.01 and serum concentration 32.60 (28.55–37.85) pg/ml vs.11.45 (9.78–12.55) pg/ml, P < 0.01] both significantly higher in AD patients than healthy controls |
| Nograles KE et al. | 2009 | Atopic dermatitis and psoriasis | **Case-control study/**  **IIIb** | 12 chronic AD patients, 13 psoriasis patients | T-cell subsets in skin biopsy and peripheral blood | - no significant differences in peripheral blood cells between AD and psoriatic patients  - TH1 and TH17 T cells significantly increased in psoriasis, TH2 T cells significantly increased in AD |
| Moy AP et al. | 2015 | Psoriasis or atopic dermatitis | **Cross sectional retrospective study/**  **IV** | 52 patients (20 psoriasis, 7 erythrodermic psoriasis, 20 AD, 50 erythrodermic AD) | Percentage of TH1, TH2, TH17, and TH22 cells in CD3+ T cells and the TH1:TH2 ratio in skin biopsies. | No significant difference in the percentage of TH1, TH2, TH17 and TH22 cells between erythrodermic psoriasis and AD. |
| Leonardi S et al. | 2015 | Atopic eczema/dermatitis syndrome (AEDS) | **Case-control study/**  **IIIb** | 104 children with atopic AEDS, 77 children with non-atopic AEDS and 93 healthy children | Serum IL-17 levels | - IL-17 significantly higher in AEDS  compared to healthy group.  - IL-17 significantly higher in aAEDS compared to naAEDS.  - positive correlation between SCORAD and IL-17  - IL-17 value positively related to total IgE levels in aAEDS.  - Further increase of IL-17 in aAEDS subjects with atopic diseases such as asthma and rhinitis compared to children with only allergic sensitization. |
| Gamez et al. | 2020 | Umbilical cord blood from atopic mothers | **Case-control study/**  **IIIb** | 7 children developed AD; 24 did not | Reduced IL-17 and IL-25 in cord blood | - reduced IL-17 and IL-25 in the seven AD infants compared to the 24 non-AD infants during the first 12 months |
